# Metagenome-assembled genomes for N_2_-fixing cyanobacterium *Nostoc* sp. TISTR 8405 and co-occurring microorganisms from a long-term laboratory culture

**DOI:** 10.1128/mra.00553-26

**Published:** 2026-08-18

**Authors:** Nannaphat Sukkasam, Tony X. Liu, Kalen Dofher, Tanakarn Monshupanee, Steven J. Hallam

**Affiliations:** 1 Department of Microbiology and Immunology, University of British Columbia 8166, Vancouver, BC, Canada; 2 Graduate Program in Bioinformatics, University of British Columbia 8166, Vancouver, BC, Canada; 3 Genome Science and Technology Program, University of British Columbia 8166, Vancouver, BC, Canada; 4 Department of Biochemistry, Faculty of Science, Chulalongkorn University 26683, Bangkok, Thailand; 5 Bradshaw Research Institute for Minerals and Mining (BRIMM), University of British Columbia 8166, Vancouver, BC, Canada; 6 Life Sciences Institute, University of British Columbia 8166, Vancouver, BC, Canada; 7 ECOSCOPE Training Program, University of British Columbia 8166, Vancouver, BC, Canada; California State University San Marcos, San Marcos, California, USA

**Keywords:** cyanobacteria, metagenome-assembled genomes, microbial consortia, nitrogen fixation, metabolic exchange, co-culture, long-read sequencing

## Abstract

We report here metagenome-assembled genomes from a long-term laboratory culture of the nitrogen-fixing cyanobacterium *Nostoc* sp. TISTR 8405, originally sourced from a Thai freshwater lake. The community consists of two additional co-occurring microorganisms, *Erythrobacter* sp. THAI-01 and *Allorhizobium* sp. THAI-01, and contains putative plasmids associated with *Nostoc* and *Allorhizobium*, respectively.

## ANNOUNCEMENT

Members of the genus *Nostoc* have emerged as versatile microbial cell factories, given their ability to convert light, water, and carbon dioxide into energy and organic material via photosynthesis while fixing atmospheric nitrogen (N_2_) (1–5). Some *Nostoc* sp. are known to live in consortia to support their growth, indicating potential for metabolic cooperation (6, 7). Here, we describe metagenome-assembled genome sequences from a long-term laboratory co-culture of *Nostoc* sp. TISTR 8405 using single-molecule long-read sequencing to identify genomic features relevant to bioproduction and microbial consortia design.

**Fig 1:**
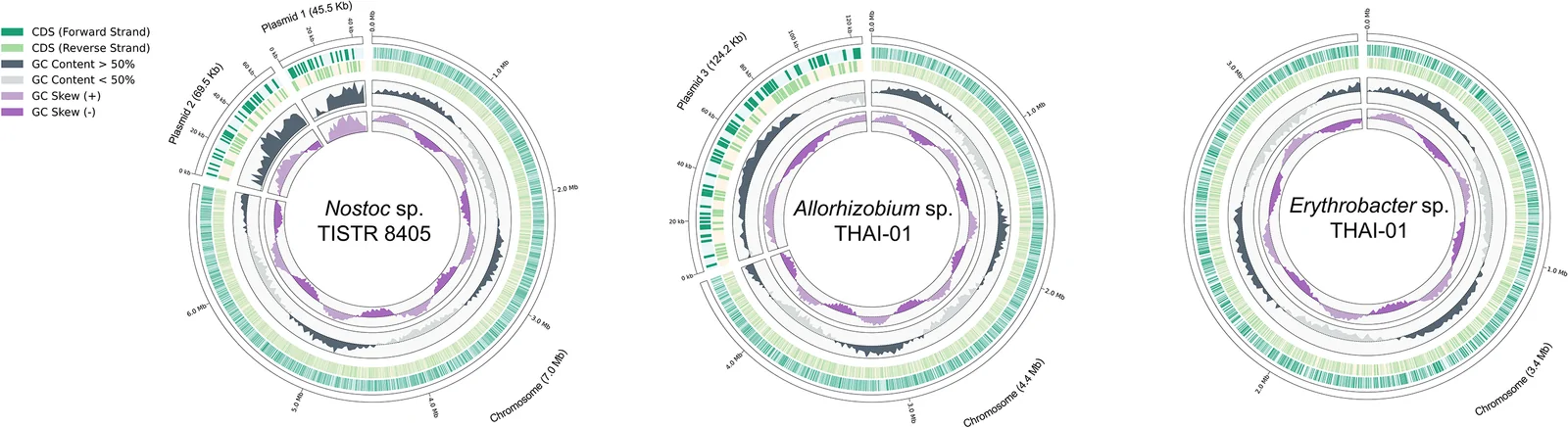
Circular maps representing metagenome-assembled genomes from the community culture. The maps display the genomic features of *Nostoc* sp. TISTR 8405, *Allorhizobium* sp. THAI-01, and *Erythrobacter* sp. THAI-01 and cognate mobile genetic elements indicated here as Plasmid 1, Plasmid 2, and Plasmid 3. From the outer to inner rings: coding sequences (CDS) on the forward strand, CDS on the reverse strand, GC content, and GC skew.

In 2001, 100-mL of source sample was collected from Nam Un (17°13′57.4″N 103°42′57.4″E), a freshwater lake in Sakon Nakhon, Thailand, and cultivated via serial dilution and colony streaking on blue-green (BG-11) medium at the Thailand Institute of Scientific and Technological Research (TISTR). The resulting co-culture was maintained in BG-11 at 26°C under continuous illumination at 50 µmol photons m⁻² s⁻¹. Genomic DNA was extracted using the Monarch high molecular weight (HMW) DNA Extraction Kit (New England Biolabs), yielding 26 ng/µL based on a NanoDrop-1000 spectrophotometer (Thermo) and a Qubit-4 Fluorometer with Qubit 1× dsDNA HS Assay Kit (Thermo) quantification. Average fragment length was 4,860 to >60,000, measured with a 4150 TapeStation system (Agilent, G2992AA). HMW DNA was sheared by pipette-tip cycling on a Microlab Nimbus (Hamilton), library prepped for the Pacific Biosciences (PacBio) platform with the Revio SPRQ HiFi Prep Kit, and size-selected on a BluePippin system (3–50 kb), generating 274,707 raw reads with a maximum length, N50, and total reads of 29,762 bp, 9,834 bp, and ~2.6 × 10^9^ bp, respectively. Statistics were calculated by SeqKit (8) (v2.12.0). Assembly of raw FASTQ reads using Hifiasm-meta (9) (v0.3.5) produced one linear and five circular contigs accounting for 99.999% of reads based on minimap2 (10) (v2.15). CheckM (11) (v1.1.3) estimated completion/redundancy of three contigs >3.4 Mb to be 99.48/0.00%, 99.48/0.54%, and 100.00/0.71%, and GTDB-Tk (12) (v2.6.1 with r226) supported classification of these contigs as *Nostoc* sp. TISTR 8405, *Erythrobacter* sp. THAI-01, and *Allorhizobium* sp. THAI-01, based on nearest-neighbor matches to *Nostoc* sp. (GCA_020829595.1), *Erythrobacter* sp. (GCF_002215495.1), and *Allorhizobium* sp. (GCA_032102365.1), respectively (Fig. 1). Further classification by Metabuli (13) (v1.1.0) matched the remaining three contigs at the family level, each <125 kb, to the above chromosomes as putative plasmids. Automated gene feature prediction and annotation of the six contigs, including genes, open reading frames, tRNAs, rRNA operons, and pseudogenes, were performed using the Prokaryotic Genome Annotation Pipeline (PGAP) (14) (Table 1). Default parameters were used unless otherwise noted.

**TABLE 1:** Features and statistics of resolved metagenome-assembled genomes. Completeness and redundancy were not measured for plasmids.

Name	NCBI accession number	PGAP ver.	Chromosome/plasmid size (bp)	GC (%)	Genes	Protein CDS	Pseudogenes	rRNA operons	tRNAs	Completeness/redundancy (%)
*Nostoc* sp. TISTR 8405	JBWBQA010000001 (CM170297)	6.11	6,953,602	40.30	5,959	5,800	72	4	71	99.48/0.00
*Anabaenopsis* Plasmid 1^*a*^	JBWBQA010000002	6.11	45,470	40.74	54	50	4	0	0	N/A^*b*^
*Trichormus* Plasmid 2	JBWBQA010000003	6.11	69,472	39.20	57	57	0	0	0	N/A
*Erythrobacter* sp. THAI-01	JBWBPZ010000001 (CM170295)	6.10	3,366,654	68.04	3,273	3,208	14	1	44	99.48/0.54
*Allorhizobium* sp. THAI-01	JBWBPY010000001 (CM170296)	6.10	4,448,840	61.20	4,329	4,240	25	3	51	100.00/0.71
*Allorhizobium* Plasmid 3	JBWBPY010000002	6.10	124,178	57.72	120	114	6	0	0	N/A

^*a*^ Plasmid 1 is represented by a linear contig. Plasmids are named after their genus-level assignments by Metabuli. Plasmids 1 and 2 were matched to the *Nostoc* sp. chromosome, while Plasmid 3 was matched to the *Allorhizobium* sp. chromosome.

^*b*^ N/A, not applicable.

Long-term co-culture stability in BG-11, which lacks exogenous organic carbon and B-vitamins, indicates the potential for metabolic cooperation. Phototrophic *Nostoc* likely serves as a primary producer, fixing both carbon and nitrogen (based on the presence of *rbc* and *nif* gene clusters), but lacks a complete cobalamin (*cob*, vitamin B12) biosynthesis pathway. Meanwhile, *Erythrobacter,* which also lacks a complete B12 biosynthesis pathway, contains unique capsular polysaccharide and expanded ROS-scavenging pathways that may contribute to biofilm stability. *Allorhizobium*, which contains a complete B12 biosynthesis pathway, has the potential to provision both *Nostoc* and *Erythrobacter* with this essential cofactor. Future studies integrating multi-omic information (RNA, protein, and metabolites) with axenic isolation are needed to validate these predictions.

## Data Availability

Plasmid sequences and associated metagenome-assembled genomes of Nostoc sp. TISTR 8405 are publicly available under NCBI accession no. JBWBQA000000000 under the BioSample SAMN54935253, Erythrobacter sp. THAI-01 under accession no. JBWBPZ000000000 under SAMN54935254, and Allorhizobium sp. THAI-01 under accession no. JBWBPY000000000 under SAMN54935255. The BioProject for all three is PRJNA1405787, and the raw reads are available from NCBI’s Sequence Read Archive (accession no. SRR37077409). Computational steps, including software dependencies, are documented on GitHub at https://github.com/hallamlab/Genome_announcements/tree/archive/nostoc_TISTR_8405_community.
